# Challenging Approach to the Development of Novel Estrogen Receptor Modulators Based on the Chemical Properties of Guaiazulene

**DOI:** 10.3390/ijms23031113

**Published:** 2022-01-20

**Authors:** Kiminori Ohta, Asako Kaise, Takumi Ogawa, Yasuyuki Endo

**Affiliations:** 1 School of Pharmacy, Showa University, 1-5-8 Hatanodai, Shinagawa-ku, Tokyo 142-8555, Japan; 2 Faculty of Pharmaceutical Sciences, Tohoku Medical and Pharmaceutical University, 4-4-1 Komatsushima, Aoba-ku, Sendai 981-8558, Japan; kaise@tohoku-mpu.ac.jp (A.K.); ogawa-takumi@pmda.go.jp (T.O.); yendo@tohoku-mpu.ac.jp (Y.E.)

**Keywords:** nuclear estrogen receptor, estrogen receptor partial agonist, azulene, tamoxifen, drug design

## Abstract

Tamoxifen, a therapeutic agent for breast cancer, has been associated with genetic polymorphisms in the metabolism of *N*,*N*-dialkylaminoethyl substituent, which plays an important role in the expression of selective estrogen receptor modulator (SERM) activity. To solve this problem, we developed a novel estrogen receptor (ER) modulator, Az-01, on the basis of the aromaticity, dipole moment, and isopropyl group of guaiazulene. Az-01 showed four-fold lower binding affinity for ER than E2 but had similar ER-binding affinity to that of 4-hydroxytamoxifen (4-HOtam). Unlike tamoxifen, Az-01 acted as a partial agonist with very weak estrogenic activity at high concentrations when used alone, and it showed potent anti-estrogenic activity in the presence of E2. The cell proliferation and inhibition activities of Az-01 were specific to ER-expressing MCF-7 cells, and no effect of Az-01 on other cell proliferation signals was observed. These findings are important for the development of new types of SERMs without the *N*,*N*-dialkylaminoethyl substituent as a privileged functional group for SERMs.

## 1. Introduction

**Figure 1:**
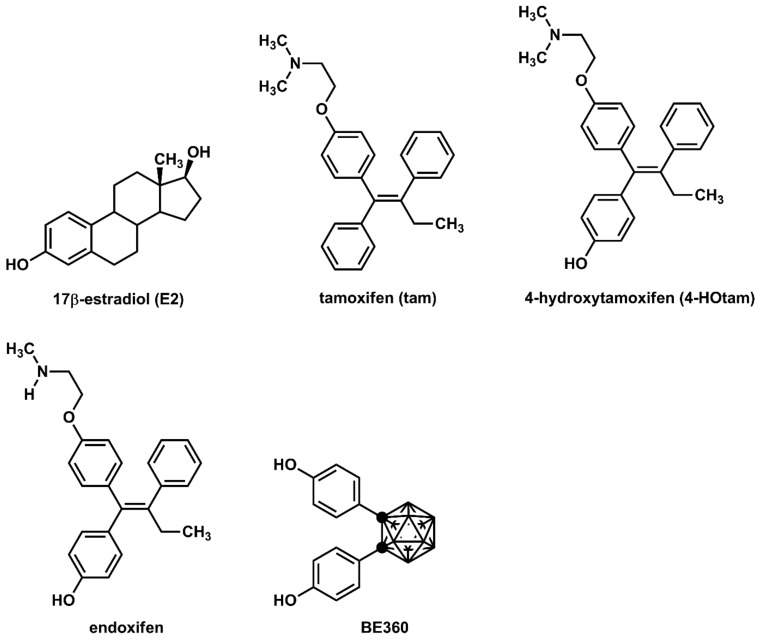
Chemical structures of various ER modulators.

Tamoxifen (tam, Figure 1), which is used worldwide for the treatment of breast cancer, competes with female hormone 17α-estradiol (E2) for binding to estrogen receptor α (ERα) [1]. Tamoxifen is a selective estrogen receptor modulator (SERM) that acts as either an agonist or an antagonist depending on tissue type [2]. It exhibits anti-estrogenic effects in breast cancer and hot flashes as well as estrogenic effects in bone and cholesterol metabolism [1,2]. Additionally, new-generation SERMs such as raloxifene and bazedoxifene have been developed to reduce the risk of side effects [3,4]. The *N*,*N*-dialkylaminoethyl substituent of these SERMs inhibits the binding of a coactivator to ERα by moving helix-12 of the ERα ligand-binding domain (LBD) to an unfavorable position [5]. Therefore, the *N*,*N*-dialkylaminoethyl substituent is regarded as a superior substituent in the development of SERMs.

Tamoxifen is metabolized in vivo to active derivatives, 4-hydroxytamoxifen (4-HOtam) and endoxifen [6,7]. These active metabolites exert more than 100 times more potent anti-estrogenic effects than tamoxifen [6,7]. Since the metabolism of tamoxifen to endoxifen involves the hepatic metabolic enzyme CYP2D6, the genetic polymorphisms of CYP2D6 have been extensively studied in relation to the potential individual variability in the efficacy of tamoxifen [8,9,10]. On the other hand, we have developed several effective ER modulators using carboranes as the hydrophobic pharmacophore [11,12,13,14]. 1,2-Bis(4-hydroxyphenyl)-*o*-carborane, hereinafter referred to as BE360, showed partial agonistic activity on ERα, acting as an agonist in bone and as an antagonist in female reproductive tissues [15]. It has been suggested that the hydrophobic structure and the dihedral angle of the two benzene rings are important for the development of ERα antagonists or ERα partial agonists.

**Figure 2:**
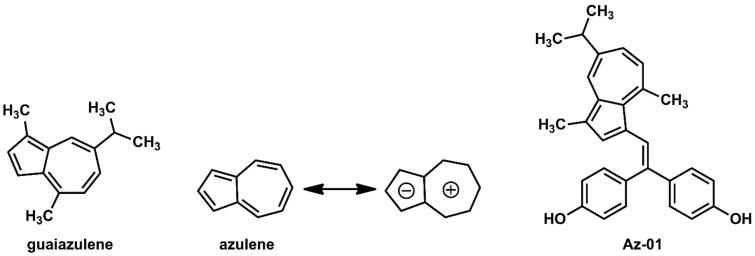
Chemical structures of guaiazulene, azulene, and novel ER modulator Az-01.

Guaiazulene and its derivatives (Figure 2) are widely used in pharmaceuticals and cosmetics because of their moderate anti-inflammatory activities [16]. Azulene, the central skeleton of guaiazulene, is a typical non-benzene aromatic compound that has a fused-ring structure composed of cyclopentadiene and cycloheptatriene [17]. Since cyclopentadiene and cycloheptatriene can be stabilized as aromatics by the 6π-electron system of the cyclopentadienyl anion and the cycloheptatrienyl cation, respectively, they have a large dipole moment (1.08 D) for hydrocarbon compounds [17]. In addition, the five-membered ring of azulene is characterized by its high electron density, which enables azulene to easily react with electrophiles particularly at 1- and 3-positions where electrophilic substitution reactions can occur [17]. We believe that azulene, which has unique and highly interesting chemical features not found in benzene systems, could be utilized as part of molecular frameworks for the development of novel drug candidates with new properties. Guaiazulene is not only safe for humans but also useful as part of a pharmaceutical structure because of the following chemical features: (1) the reaction site can be easily controlled by the substituents; (2) the three hydrophobic substituents enable hydrophobic interactions with the target molecule; and (3) the bulky isopropyl group provides steric repulsion with the amino acid residues of the target molecule.

Considering the interaction between guaiazulene and ERα LBD, it was expected from the dipole moment of azulene that guaiazulene would prefer not only the hydrophobic pocket of ERα but also the space where helix-12 existed. Furthermore, it was expected that the steric hindrance caused by the isopropyl group would also inhibit the folding of helix-12. Therefore, on the basis of the chemical structures of 4-HOtam and BE360, we designed Az-01 as a novel ER modulator without the *N*,*N*-dialkylaminoethyl substituent (Figure 2).

## 2. Results and Discussion

**Scheme 1:**
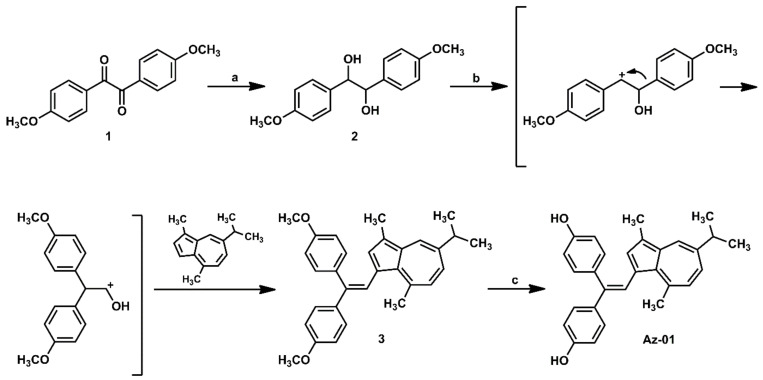
Synthesis of azulene-containing ER modulator candidate Az-01. *Reagents and conditions:* (**a**) NaBH_4_, THF, MeOH, rt, quantitative; (**b**,**c**). HCl, MeOH, 60 ^o^C, 99%; (**c**) BBr_3_, CH_2_Cl_2_, room temperature, quantitative.

The synthesis of the designated azulene derivative Az-01 is summarized in Scheme 1. Reduction of the two adjacent carbonyl groups of 4,4′-dimethoxybenzyl (**1**) with sodium borohydride (NaBH_4_) at room temperature gave dihydroanisoin (**2**) quantitatively as a diastereoisomer. When diastereomixture **2** was reacted with guaiazulene under the reaction conditions reported by Nakatsuji et al., the carbocation intermediate generated by the pinacol rearrangement of **2** and an electron-rich five-membered ring of guaiazulene underwent an electrophilic substitution reaction followed by dehydration to give trisubstituted alkene (**3**) in 99% yield [18]. The demethylation of **3** using BBr_3_ gave Az-01 quantitatively, which was identified by matching its ^1^H NMR spectra with those previously reported for Az-01 [18].

**Figure 3:**
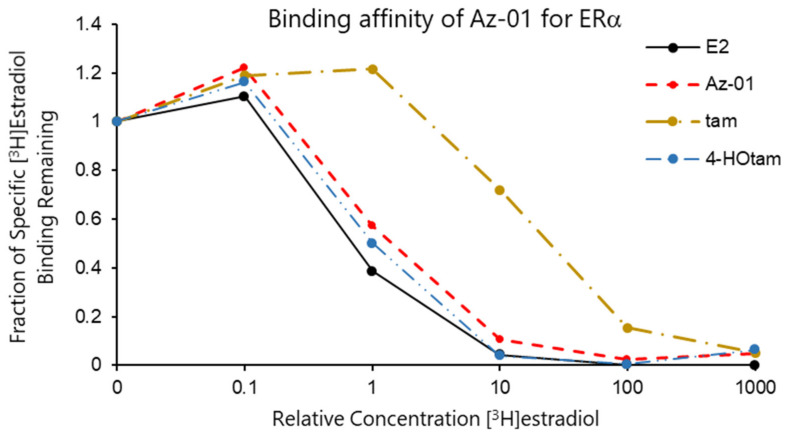
Assay for competitive binding of Az-01, tam, and 4-Hotam with [^3^H]estradiol at Erα LBD. Binding assays were performed using the test compounds and unlabeled E2 (0.4–4 μM) in the presence of [2,4,6,7-^3^H]17β-estradiol (4 nM) in duplicate.

The binding affinities of Az-01, tam, and 4-HOtam for ERα LBD were evaluated by carrying out a competitive binding assay using human recombinant ERα and [2,4,6,7-^3^H]17β-estradiol (radiolabeled E2) [19]. The test compounds were incubated with 4 nM [^3^H]E2 in the presence of ERα LBD at 4 °C for 18 h, which was followed by the filtration and measurement of radioactivity produced by the complexation of ERα LBD with [^3^H]E2 on the nitrocellulose membrane. All the test compounds competed with [^3^H]E2 and were bound to ERα in a concentration-dependent manner. Figure 3 shows the binding affinity of non-radioactive E2 as a control and Az-01, tam, and 4-HOtam for ERα. The horizontal axis of the graph shows the ratio of the concentrations of test compound to [^3^H]estradiol (non-radioactive compound/[^3^H]estradiol), and the vertical axis shows the specific activity of [^3^H]estradiol bound to ERα remaining on the nitrocellulose membrane. The binding affinity of each test compound was calculated from the radioactivity of radiolabeled E2 bound to ERα. The IC_50_ value of each test compound was calculated from the concentration-dependent sigmoidal curve obtained from the competitive binding assay. The IC_50_ values of Az-01, tam, and 4-HOtam were 1.22 nM, 27.7 nM, and 0.84 nM, respectively. When the binding affinity of non-radioactive E2 was taken as 100%, the relative binding affinities (RBAs) of Az-01, tam, and 4-HOtam were 26%, 1.1%, and 38%, respectively. The RBA value of Az-01 was approximately four-fold lower than that of E2 and similar to that of 4-HOtam. The much lower RBA of tam than those of E2, Az-01, and 4-HOtam was due to the absence of a phenolic hydroxy group. In general, estrogen receptor antagonists such as 4-HOtam inhibit the formation of transcription factor complexes when a part of their chemical structures comes in contact with helix-12 of the receptors. Therefore, their chemical structures tend to be bulky, and their affinities for the receptors are lower than those of the corresponding native ligands. The guaiazulene structure of Az-01 may make it difficult for Az-01 to interact with the hydrophobic pocket of Erα LBD because of the high polarization of azulene and the presence of bulky substituents. From the comparison of the binding affinities of Az-01 and 4-Ohtam for Erα, we considered that Az-01 had sufficiently high binding affinity as an ER modulator. These results indicated that the azulene ring would be very useful as a substructure of ER modulators, and the results obtained here would provide vital information for the development of novel types of ER modulators.

**Figure 4:**
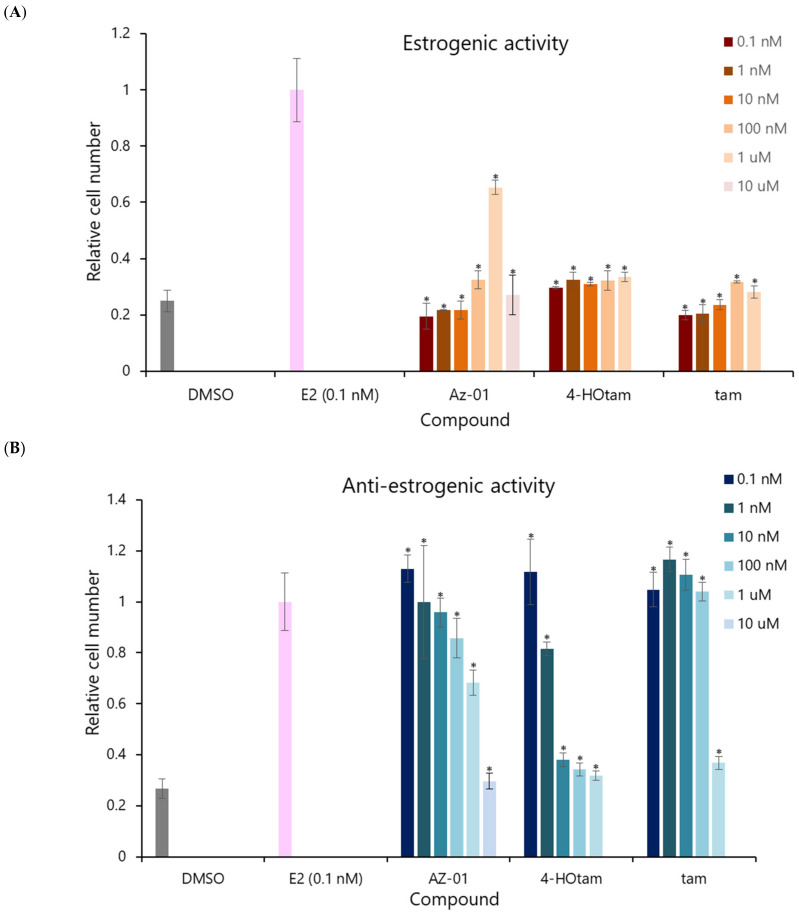
Estrogenic and anti-estrogenic activities of Az-01, 4-hydroxytamoxifen, and tamoxifen in MCF-7 cell proliferation assay. (**A**) Estrogenic activity of the test compounds. (**B**) Anti-estrogenic activity of test compounds on E2-induced MCF-7 cell proliferation. The test compounds were prepared in DMSO solution by making a series dilution so that the final concentration of DMSO was 0.5%. E2 was used at the lowest concentration (0.1 nM) that showed the highest estrogenic activity, as determined from the concentration-dependent curve. All assays were performed using sFBS from which various growth factors had been removed. The vertical axis shows the ratio of the number of proliferating cells in the presence of each test compound to the number of cells in the presence of 0.1 nM E2 taken as 1. All assays were performed in triplicate (*n* = 3). The two-tailed Student’s *t*-test was used for statistical analyses (* *p* < 0.001).

Next, we evaluated the estrogenic activity of Az-01 by performing the cell proliferation assay using the human breast adenocarcinoma cell line MCF-7, which shows estrogen-dependent growth (Figure 4) [19]. To confirm the direct effect of the test compounds on Erα, sFBS, from which lipophilic growth factors had been removed, was used in the assay [20]. The ER-dependent proliferative and anti-proliferative activities of the test compounds were measured at concentrations ranging from 1 × 10^−10^ M to 1 × 10^−5^ M. DMSO was used as control for estrogenic activity and E2 was used as control for anti-estrogenic activity. Tam and 4-Hotam were also used as comparative and positive controls in this assay. The number of viable cells was calculated from an assay using water-soluble formazan (WST-8). Neither 4-Hotam nor tam showed estrogenic (ER agonistic) activity when the compounds were used alone (Figure 4A). Although Az-01 did not show estrogenic activity at concentrations below 10 nM, it exhibited concentration-dependent estrogenic activity at concentrations of 100 nM to 1 μM. Moreover, Az-01 at 10 μM showed no estrogenic activity. When the ER-dependent cell proliferation effect of E2 at 0.1 nM was set at 100%, the maximum estrogenic activity shown by Az-01 was approximately 70% (1 μM). Although Az-01 showed potent binding affinity for Erα LBD, its estrogenic activity was very weak. Next, we examined the effect of these test compounds on the estrogen-dependent cell proliferation effect of 0.1 nM E2 (Figure 4B). Tam inhibited the proliferation of MCF-7 cells at 1 μM. In addition, its active metabolite, 4-Hotam, had more than 100-fold more potent anti-estrogenic activity than tam and completely inhibited E2-induced MCF-7 cell proliferation at 10 nM. Az-01 inhibited the estrogenic effect of E2 in a concentration-dependent manner, but the inhibitory effect was not complete, and the inhibition was approximately 70% of the cell proliferation induced by E2 at 0.1 nM. Az-01, which lacked the *N*,*N*-dialkylaminoethyl substituent, acted as an ER partial agonist. The observed antagonism of Az-01 to E2 despite its weak estrogenic activity might be due to Az-01 competing with E2 for binding to ERα LBD. Since the anti-estrogenic activity of Az-01 at 1 μM was comparable to the estrogenic activity of Az-01 used alone at the same concentration, it seemed that the activity at this concentration was entirely derived from Az-01. Treatment with Az-01 at 10 μM completely inhibited the estrogenic activity of E2 similarly to 4-HOtam. We speculated that the chemical structure of Az-01 well fit the hydrophobic space of ERα LBD, but part of its structure hindered the complex formation between ERα and related transcription factors. These results are very interesting for the development of new types of ER modulators, and an understanding of the binding mode of Az-01 to ERα LBD will lead to the discovery of SERMs with novel chemical structures.

**Figure 5:**
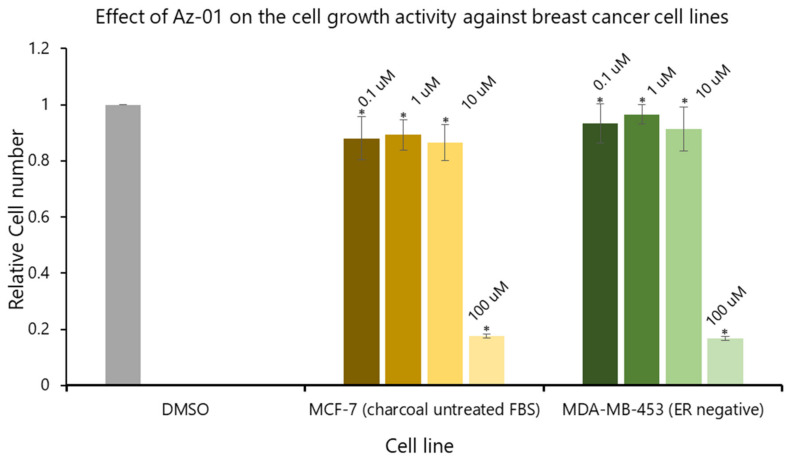
Effect of Az-01 on the growth of MCF-7 cells (ER-positive) and MDA-MB-453 cells (ER-negative). Az-01 was prepared in DMSO solution by making a series dilution so that the final concentration of DMSO was 0.5%. All assays were performed using activated-charcoal-untreated FBS. All assays were performed in triplicate (*n* = 3). The two-tailed Student’s t-test was used for statistical analyses (* *p* < 0.001).

To clearly understand the ER-mediated partial agonistic activity of Az-01, we evaluated the effect of Az-01 on MCF-7 cell growth using activated-charcoal-untreated FBS. We also examined the effect of Az-01 on the growth of MDA-MB-453 cells that do not express ER. Figure 5 summarizes the effects of Az-01 on the growth of both cell lines. By using activated-charcoal-untreated FBS, we found that Az-01 did not induce MCF-7 cell growth even at the high concentration of 10 μM. In addition, Az-01 did not inhibit the growth of ER-negative MDA-MB-453 cells. On the other hand, treatment with Az-01 at an even higher concentration of 100 μM induced cell death in both cell lines. In the ER-dependent cell proliferation study using MCF-7 cells (Figure 4), Az-01 was used at concentrations that did not induce cytotoxicity. Therefore, the results in Figure 4 clearly showed that Az-01 had partial agonistic activity through the disruption of ER-dependent cell proliferation signals. When considering cytotoxicity, it would be appropriate to use Az-01 at concentrations below 10 uM.

**Figure 6:**
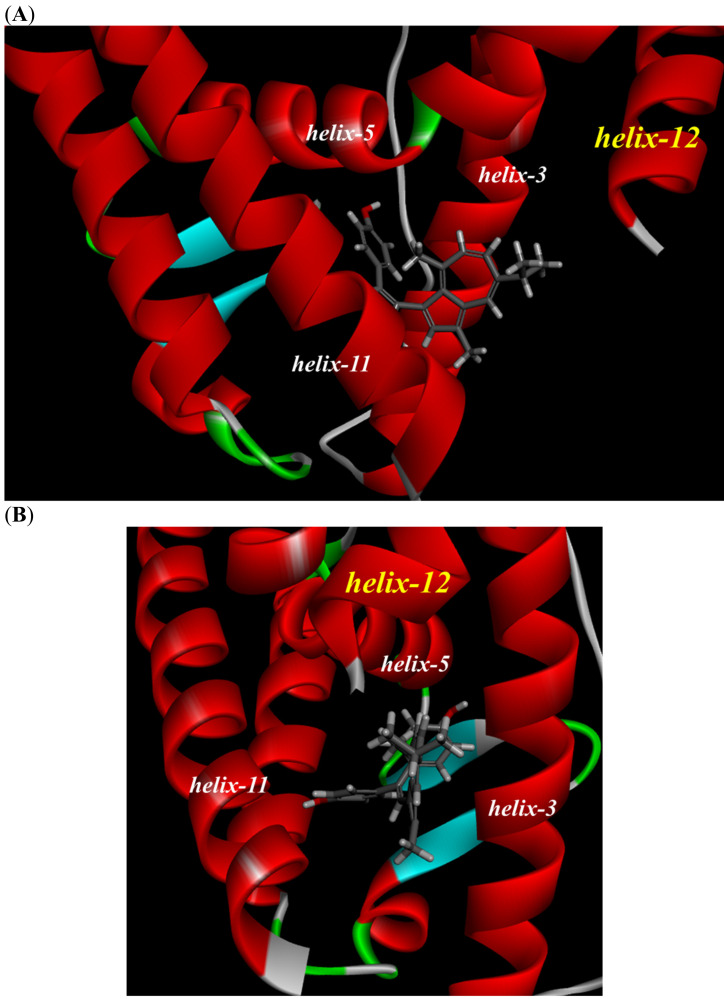

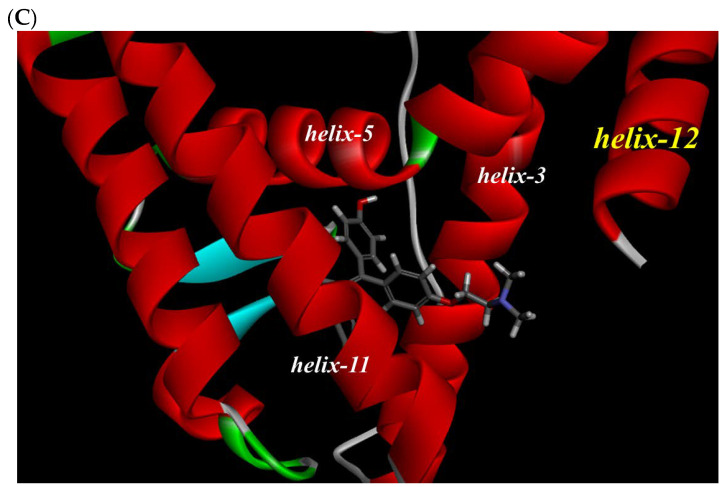
Docking modes of Az-01 and 4-HOtam in ERα LBD. (**A**,**B**) Binding modes of Az-01. (**C**) Binding mode of 4-HOtam.

To investigate the reason for the partial agonistic activity of Az-01, we carried out docking simulation studies of Az-01 with ERα LBD. By using the cocrystal structure of ERα LBD with raloxifene obtained from the RCSB Protein Data Bank (PDBID: 1ERR) [21], we calculated the binding modes using Discovery Studio 2019 equipped with the CHARMm molecular simulation program [22]. The test compounds were conformationally optimized using the CHARM force field. The calculated results for Az-01 and 4-HOtam with ERα LBD are shown in Figure 6. The guaiazulene moiety of Az-01 was not accommodated in the hydrophobic pocket of ERα but was located in the space where helix-12 of ERα existed (Figure 6A). In addition, the isopropyl group of the guaiazulene moiety was close to helix-12 of ERα (Figure 6B). This proximity between the isopropyl group and helix-12 would be attributed to the ER partial agonistic activity. Figure 6C shows the docking mode of 4-HOtam with ERα; the alkylamino group of 4-HOtam was located close to helix-12. The electrostatic interaction between the alkylamino group of 4-HOtam and the amino acid residue of Asp351 in helix-3 was important for the ER antagonistic activity. We speculated that the cationic seven-membered ring of Az-01 would show similar electrostatic interaction to the alkylamino group of 4-HOtam. Since the cationic seven-membered ring also polarized the substituents attached to it, the structural transformation of the azulene moiety of Az-01 would be used to develop more potent ER full antagonists that would be more potent than 4-HOtam. Furthermore, if carborane, which we are currently studying as a new hydrophobic pharmacophore, could be used as the hydrophobic structure, improvement of the binding affinity of Az-01for ERα LBD would be highly expected.

## 3. Materials and Methods

### 3.1. Synthesis of Az-01

^1^H NMR spectra were recorded with JEOL JNM-LA-400 spectrometers (Tokyo, Japan), and chemical shifts were referenced to tetramethylsilane (0.0 ppm) as the internal standard. The splitting patterns were designated as follows: s (singlet), d (doublet), sept (septet), and m (multiplet). Column chromatography was carried out using Fuji Silysia silica gel BW-80S (Fuji Silysia, Kasugai, Japan), and TLC was performed on Merck silica gel F254 (Merck, Tokyo, Japan). Reagents were purchased from FUJIFILM Wako Pure Chemical Corp. (Osaka, Japan), Kanto Chemical Ltd. (Tokyo, Japan), and Tokyo Chemical Industry Ltd. (Tokyo, Japan). All solvents were of reagent grade, purchased commercially, and were used without further purification.

#### 3.1.1. 4,4′-Dihydroanisoin (**2**)

To a solution of 4,4′-dimethoxybenzil (**1**) (2.72 g, 10 mmol) in 10 mL of THF and 10 mL of MeOH was added NaBH_4_ (760 mg, 20 mmol) at room temperature, and the reaction mixture was stirred for 2 h at the same temperature. The reaction mixture was poured into water and extracted with AcOEt. The extract was dried over MgSO_4_ and concentrated to obtain **2** (2.77 g, 10.1 mmol, quant.) as a colorless solid.

^1^H NMR (400 MHz, DMSO-*d_6_*) *δ* (ppm) 3.29 (s, 6 H), 4.47 (d, *J* = 3.4 Hz, 2 H), 4.98 (d, *J* = 3.4 Hz, 2 H), 6.78 (d, *J* = 8.7 Hz, 4 H), 7.11 (d, *J* = 8.7 Hz, 4 H).

#### 3.1.2. 3-[2,2-Bis(4-methoxyphenyl)vinyl]-7-isopropyl-1,4-dimethylazulene (**3**)

To a solution of guaiazulene (500 mg, 2.52 mmol) in 10 mL of MeOH was added a solution of **2** (600 mg, 2.19 mmol) in 15 mL of MeOH containing 36% HCl aqueous solution (2 mL) at 60 °C. The reaction mixture was stirred at the same temperature for 5 h under aerobic conditions, poured onto ice, and then extracted with CH_2_Cl_2_. The organic layer was washed with brine, dried over MgSO_4_, and concentrated. The residue was purified by silica gel column chromatography (eluent: *n*-hexane/AcOEt = 10/1 to 1/2) to obtain **3** (950 mg, 2.17 mmol, 99%) as a green solid.

^1^H NMR (400 MHz, CDCl_3_) *δ* (ppm) 1.31 (s, 3 H), 1.32 (s, 3 H), 2.37 (s, 3 H), 2.96 (sept, *J* = 6.8 Hz, 1 H), 3.01 (s, 3 H), 3.80 (s, 3 H), 3.83 (s, 3 H), 6.76 (d, *J* = 8.7 Hz, 2 H), 6.79 (d, *J* = 11.1 Hz, 1 H), 6.86 (d, *J* = 9.2 Hz, 2 H), 6.97 (s, 1 H), 7.15 (d, *J* = 8.7 Hz, 2 H), 7.14–7.20 (m, 1 H), 7.29 (d, *J* = 7.7 Hz, 2 H), 7.49 (s, 1 H), 7.88 (d, *J* = 1.9 Hz, 1 H).

#### 3.1.3. 4,4′-(2-(5-Isopropyl-3,8-dimethylazulen-1-yl)ethene-1,1-diyl)diphenol (Az-01)

To a solution of **3** (950 mg, 2.17 mmol) in 10 mL of CH_2_Cl_2_ was added 9 mL of 1 M of BBr_3_ solution in CH_2_Cl_2_ (9 mmol) at room temperature, and the reaction mixture was stirred for 4 h at the same temperature. The reaction mixture was poured into water and extracted with AcOEt, and the extract was dried over MgSO_4_ and concentrated. The residue was purified by silica gel column chromatography (eluent: *n*-hexane/AcOEt = 5/1 to 3/1) to obtain **3** (900 mg, 2.21 mmol, quant.) as a green solid.

Green prisms (AcOEt/*n*-hexane); ^1^H NMR (400 MHz, C_6_D_6_) *δ* (ppm) 1.14 (s, 3 H), 1.15 (s, 3 H), 2.28 (s, 3 H), 2.68 (sept, *J* = 6.8 Hz, 1 H), 2.88 (s, 3 H), 3.87 (s, 1 H, D_2_O exchangeable), 3.96 (s, 1 H, D_2_O exchangeable), 6.39 (d, *J* = 8.7 Hz, 2 H), 6.51 (d, *J* = 8.7 Hz, 2 H), 6.59 (d, *J* = 10.6 Hz, 2 H), 6.92–7.00 (m, 1 H), 7.24 (d, *J* = 8.7 Hz, 2 H), 7.33 (d, *J* = 8.2 Hz, 2 H), 7.37 (s, 1 H), 7.68 (s, 1 H), 7.88 (d, *J* = 1.9 Hz, 1 H).

### 3.2. Docking Simulation Studies of Az-01 with ERα

Docking simulation studies were performed using the three-dimensional structure of the ERα-raloxifene complex (PDB ID: 1ERR) obtained from the RCSB Protein Data Bank using Discovery Studio 2019 software (BIOVIA, San Diego, CA, USA). Calculations were performed after the ligand raloxifene was removed from the complex structure and replaced with the conformationally optimized test compounds using the CHARMm force field. The missing hydrogen atoms in the complex structure were added to the optimal positions by calculation.

### 3.3. Competitive Erα-Binding Assay

Ligand-binding on ERα was determined by the filtrate binding assay using a nitrocellulose membrane. ERα (0.5 μg/tube, Invitrogen, Waltham, MA, USA) was diluted with binding assay buffer containing 20 mM Tris–HCl (pH 8.0, Invitrogen), 0.2 mM phenylmethylsulfonyl fluoride (Wako), 1 mM EDTA (pH 8.0, Gibco), 0.3 M NaCl, and 10 mM 2-mercaptoethanol (Wako). The resulting solution was incubated with 4 nM [2,4,6,7-^3^H]17β-estradiol (Perkin Elmer, Waltham, MA, USA) in the presence or absence of the unlabeled test compound (competitor) in a microtube (1.5 mL) at 4 °C for 18 h. The incubated mixture was transferred onto a nitrocellulose membrane and filtered under reduced pressure. The ligand–ERα complex remaining on the nitrocellulose membrane was washed twice with buffer (20 mM Tris–HCl pH 8.0, 0.15 M NaCl) and then with 25% ethanol in distilled water. The radioactivity of the complex that remained on the membrane was measured in Atomlight (Perkin Elmer, Waltham, MA, USA) using a liquid scintillation counter.

### 3.4. Assay for MCF-7 Cell Proliferation

MCF-7 cells, a human breast adenocarcinoma cell line, were cultivated in DMEM supplemented with 10% FBS, 100 IU/mL penicillin, and 100 μg/mL streptomycin at 37 °C in a 5% CO_2_ humidified incubator. The day before the assay, the medium used for MCF-7 cell culture was changed to phenol-red-free, low-glucose DMEM supplemented with 5% dextran-coated activated-charcoal-stripped FBS (sFBS), 100 IU/mL penicillin, and 100 μg/mL streptomycin. After the cells were collected from the maintenance dish with phenol-red-free 0.25% trypsin-EDTA, they were seeded on a 96-well plate at the density of 2000 cells per final volume of 100 μL of phenol-red free, low-glucose DMEM supplemented with 5% sFBS, 100 IU/mL penicillin, and 100 μg/mL streptomycin. After incubation for 24 h, the medium was replaced with 90 μL of fresh phenol-red-free, low-glucose DMEM supplemented with 5% sFBS, 100 IU/mL penicillin, and 100 μg/mL streptomycin. Moreover, 10 μL of serial dilutions of the test compounds dissolved in DMSO or DMSO alone (dilution control) was added to triplicate cultures in the presence or absence of 0.1 nM estradiol. Cells for the assay conditioned by the above method were incubated for 5 days. Finally, to count the number of cells, 10 μL of Cell Counting Kit-8 (Dojindo, Kumamoto, Japan) was added to each well, which was followed by incubation for 2 h. The absorbance at 450 nm of each well was measured. This parameter indicates the number of living cells in the culture.

### 3.5. Assays for MCF-7 and MDA-MB-453 Cell Growth

Cells from the human breast adenocarcinoma cell line MCF-7 were cultivated in MEMα supplemented with 5% FBS, 100 IU/mL penicillin, and 100 mg/mL streptomycin at 37 °C in a 5% CO_2_ humidified incubator. Cells from the human breast cancer cell line MDA-MB-453 were cultivated in RPMI-1640 supplemented with 10% FBS, 100 IU/mL penicillin, and 100 mg/mL streptomycin at 37 °C in a 5% CO_2_ humidified incubator. Cells were trypsinized from the maintenance dish with trypsin-EDTA and seeded in a 96-well plate at the density of 3000 cells per well (final volume: 100 μL) for cell culture using the same medium. After 1 day, the medium was removed, and 100 μL of serial dilutions of the test compounds or DMSO (dilute control) was added to microcultures in triplicate. The cells were incubated for 3 days, at the end of which cell proliferation was evaluated using the 5 μM Cell Counting Kit-8, which was added to the microcultures before the cells were incubated for 4 h. Subsequently, the absorbance at 450 nm was measured.

## 4. Conclusions

In a challenging study aimed at developing novel types of SERMs, we have developed a new SERM candidate, Az-01, that lacks the *N*,*N*-dialkylaminoethyl substituent on the basis of the chemical features of azulene. Az-01 showed potent ERα-binding affinity and ER partial agonistic activity. The results indicated that Az-01 acted as an ER antagonist in the presence of E2 and as a weak ER agonist in the absence of E2. Docking simulation studies of Az-01 with ERα LBD suggested that the isopropyl group of the guaiazulene moiety in Az-01 was close to helix-12 in ERα LBD. We speculated that the polarization of the azulene structure in Az-01 prevented the guaiazulene moiety from fitting into the hydrophobic pocket of ERα LBD. Therefore, Az-01 would be a promising SERM candidate as an ER partial agonist. We are working on the discovery of SERMs using Az-01 as the lead compound. These findings are very useful for the development of novel types of SERMs without the *N*,*N*-dialkylaminoethyl substituent.

## Data Availability

The data presented in this study are available on request from the corresponding author.
